# How MCM loading and spreading specify eukaryotic DNA replication initiation sites

**DOI:** 10.12688/f1000research.9008.1

**Published:** 2016-08-24

**Authors:** Olivier Hyrien

**Affiliations:** 1 Institut de Biologie de l'Ecole Normale Superieure (IBENS), Ecole Normale Superieure, PSL Research University, Paris, France

**Keywords:** MCM loading, eukaryote, DNA replication, origins

## Abstract

DNA replication origins strikingly differ between eukaryotic species and cell types. Origins are localized and can be highly efficient in budding yeast, are randomly located in early fly and frog embryos, which do not transcribe their genomes, and are clustered in broad (10-100 kb) non-transcribed zones, frequently abutting transcribed genes, in mammalian cells. Nonetheless, in all cases, origins are established during the G1-phase of the cell cycle by the loading of double hexamers of the Mcm 2-7 proteins (MCM DHs), the core of the replicative helicase. MCM DH activation in S-phase leads to origin unwinding, polymerase recruitment, and initiation of bidirectional DNA synthesis. Although MCM DHs are initially loaded at sites defined by the binding of the origin recognition complex (ORC), they ultimately bind chromatin in much greater numbers than ORC and only a fraction are activated in any one S-phase. Data suggest that the multiplicity and functional redundancy of MCM DHs provide robustness to the replication process and affect replication time and that MCM DHs can slide along the DNA and spread over large distances around the ORC. Recent studies further show that MCM DHs are displaced along the DNA by collision with transcription complexes but remain functional for initiation after displacement. Therefore, eukaryotic DNA replication relies on intrinsically mobile and flexible origins, a strategy fundamentally different from bacteria but conserved from yeast to human. These properties of MCM DHs likely contribute to the establishment of broad, intergenic replication initiation zones in higher eukaryotes.

## Introduction

We review here recent progress in understanding how MCM proteins, which form the core of the eukaryotic replicative helicase, are loaded onto chromatin and redistributed along the genome to specify the location and activation time of eukaryotic DNA replication initiation sites. DNA replication is required for the faithful transmission of genetic information from mother to daughter cells ^1^. The selection of replication initiation sites (origins) ^2^ is developmentally regulated in metazoan cells ^3– 7^, with possible consequences on developmental programs ^8^ and genome stability ^9, 10^. Replication errors due to endogenous or exogenous causes can lead to cancer or genetic diseases ^11– 13^, and several DNA replication proteins including MCMs are used as cancer biomarkers ^14^. Conversely, many efficient antibacterial, antiviral, or anticancer drugs act by targeting DNA replication ^1, 15– 18^. Finally, origin selection influences the fate of DNA introduced into cells for biotechnological or therapeutic purposes or during natural DNA transfer processes. Understanding the control of replication initiation is therefore a fundamental endeavor critical to genome manipulation and to the understanding and treatment of human disease. We summarize basic mechanisms of replication initiation in bacteria and eukaryotes and then elaborate on why eukaryotic replication origins are fundamentally different from, and more flexible than, those of bacteria.

## The bacterial model for replication fork assembly

**Figure 1.**
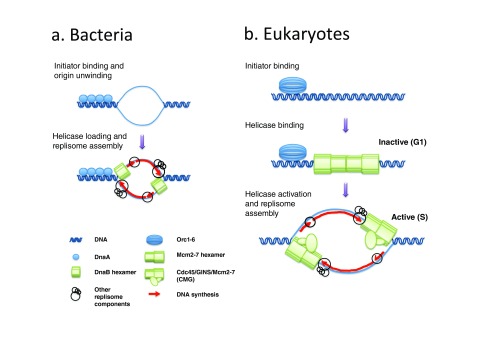
Replication initiation in bacteria ( **a**) and eukaryotes ( **b**). In bacteria ( **a**), the binding of the initiator (DnaA) to the replication origin leads to DNA melting (top). The initiator then recruits the replicative DNA helicase (DnaB) in an active form around single-stranded DNA (ssDNA). This is followed by replisome assembly and the start of DNA synthesis. Since DnaB translocates in the 5' to 3' direction along the DNA, it encircles the lagging-strand template. In eukaryotes ( **b**), the initiator (Orc1-6) loads the replicative DNA helicase (Mcm2-7) in an inactive, double-hexameric form around double-stranded DNA (dsDNA) during the G1-phase. Activation of the helicase is temporally separated from helicase loading and only occurs during S-phase by recruitment of Cdc45 and GINS to form the active Cdc45/Mcm2-7/GINS (CMG) holo-helicase. Since CMG translocates in the 3' to 5' direction along the DNA, it encircles the leading strand template.

With the exception of RNA viruses, all living organisms use DNA to encode their genetic information, and all replicate it by the replication fork mechanism, in which the two DNA strands are separated by a replicative helicase then copied by DNA polymerases ^1^. In bacterial chromosomes, strand separation typically initiates at a single site, called the replication origin, through binding of a protein factor called the initiator (DnaA in *Escherichia coli*, Figure 1a) ^19^. The initiator recognizes and unwinds origin DNA, then together with DnaC loads two inversely oriented copies of a ring-shaped homohexameric replicative helicase (DnaB _6_) around single-stranded DNA (ssDNA). The ability of each helicase complex to translocate in one direction along ssDNA, and to recruit RNA primases, DNA polymerases, and accessory factors, converts the origin DNA into two replication forks that travel and replicate DNA in opposite directions. The multiprotein complex that powers the replication fork is referred to as the replisome.

**Figure 2.**
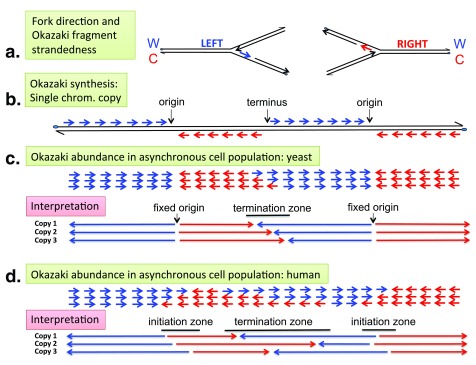
Okazaki fragment sequencing delineates initiation and termination sites. ( **a**) Relationship between replication fork direction and Okazaki fragment strandedness. Leftward- and rightward-moving forks synthesize the Watson (W) and the Crick (C) strand, respectively, in the form of Okazaki fragments (short red and blue arrows). ( **b**) Expected strandedness of Okazaki fragment synthesis along a single copy of a chromosomal DNA segment containing the indicated origins and terminus. Note that Okazaki fragments (∼0–200 nt) have been drawn to a much larger size than proportional to replication units (∼40 kb in yeast, ∼200 kb in human cells). ( **c**, **d**) Typical abundance pattern of Crick (red) and Watson (blue) Okazaki fragments (short arrows) in asynchronously growing yeast ^33, 119^ ( **c**) and human ^59^ ( **d**) cell populations ( **c**, **d**, top) and interpretation ( **c**, **d**, bottom). Long arrows indicate the movement of replication forks in individual chromosomal copies. Watson-to-Crick shifts in Okazaki fragment strandedness, diagnostic of initiation events, are abrupt in yeast ( **c**), indicating efficient usage of site-specific origins, but gradual in human cells ( **d**), indicating inefficient usage of multiple, dispersed origins within a broad zone (10–100 kb). Crick-to-Watson shifts, indicative of termination events, are gradual in both yeast and human cells, indicating that termination is dispersive in the cell population owing to a variable position and/or firing time of adjacent origins and/or a variable rate of fork progression. ****Yeast termination zones are most often relatively confined, clearly separated from origins by zones of unidirectional fork progression. In contrast, human termination zones are much broader and frequently contiguous with initiation zones.

In the replication fork mechanism, DNA synthesis occurs concomitantly on both template strands as they are unwound. Since the two template strands are antiparallel and DNA polymerases synthesize DNA only in the 5' to 3' direction, the direction of synthesis on one template must be opposite to that of fork movement ( Figure 1). This is accomplished by the repeated initiation of short RNA primed nascent DNA chains, referred to as "Okazaki fragments", that eventually join the 5' ends of long nascent DNA chains. When DNA replication proceeds bidirectionally from specific sites, a transition from discontinuous to continuous synthesis occurs across the origin. Okazaki fragments are complementary to one template strand on one side of the origin and to the other template strand on the other side ( Figure 1, Figure 2a).

Archaeal chromosomes can replicate from a single origin or multiple origins using a machinery that is much more closely related to that of eukaryotes than to that of bacteria ^20^. Archaeal ^20^ and bacterial ^19^ DNA replication initiation has been reviewed elsewhere and will not be discussed here further.

## Identification of eukaryotic DNA replication origins

Eukaryotic chromosomes contain multiple replication origins that are activated (fire) at different times during S-phase ^2, 21, 22^. Eukaryotic origins were first isolated from budding yeast as short (100–200 bp) DNA sequence elements that are able to promote autonomous plasmid replication ^23– 25^. Named ARSs (autonomously replicating sequences), these elements were shown by physical analysis of replicating DNA to coincide (at a ∼1 kb resolution) with replication initiation sites in yeast plasmids and chromosomes ^26– 34^. Yeast ARSs require two separate elements for function, a degenerate T-rich ARS consensus sequence (ACS) and an A-rich nucleosome-excluding sequence downstream of the ACS ^35, 36^. The ACS is bound by the origin recognition complex (ORC), a conserved heterohexameric AAA+ ATPase required for replication initiation in all tested eukaryotes ^37– 43^. The nucleosome-free region (NFR) adjacent to the ORC binding site is believed to provide space for the association of additional replication factors (see below). High-resolution analysis of leading strand synthesis at *ARS1* identified a single start site for each leading strand within this NFR ^44^.

In contrast to yeast, autonomous replication assays failed to isolate metazoan ARSs. Plasmid replication does not require any specific DNA sequence and initiates at random sequences in *Xenopus* eggs ^45– 47^ or in cultured mammalian cells ^48, 49^. Consistently, metazoan ORCs do not show any DNA binding sequence preference *in vitro* ^50– 52^. Replication of metazoan chromosomes initiates at random sequences in early fly ^53^ and frog ^54^ embryos, in which the genome is transcriptionally silent. In later embryos, however, the onset of zygotic transcription is accompanied by a circumscription of replication initiation to intergenic zones consisting of multiple inefficient initiation sites ^5, 6^. Broad replication initiation zones delimited by active transcription units were also observed at a few loci in mammalian somatic cells ^2, 55– 58^.

Recently, Okazaki fragment sequencing has been used to measure replication fork directionality (RFD) and delineate initiation and termination sites genome-wide in budding yeast ^33^ and in human cells ^59^ ( Figure 2). Okazaki fragment sequences indicate their strandedness ( Figure 2a) and therefore the direction of their fork of origin ( Figure 2b). This allows one to determine the frequency with which a locus is replicated rightward or leftward in a cell population ( Figure 2c, 2d). In yeast, abrupt left-to-right RFD inversions typical of punctate initiation sites are observed at consistent locations with pre-existing origin identifications ^33^ ( Figure 2c). In human cells, however, smooth leftward-to-rightward RFD inversions are observed, revealing thousands of broad (10–100 kb) replication initiation zones ^59^ ( Figure 2d). About one-half of the replication initiation zones are bordered by active transcription units on one or both sides, and these fire early in S-phase. The remainder are scattered in large non-expressed portions of the genome and fire predominantly late in S-phase. The mechanism specifying the boundaries of initiation zones in the absence of flanking active genes is unclear, although both types of initiation zones share open chromatin marks typical of active or poised enhancers ^59^. Termination occurs over broad zones of rightward-to-leftward RFD inversion in both yeast ^33^ and human ^59^ cells, at positions dictated by the firing time distributions of flanking origins ( Figure 2c, 2d).

The broad and gradual changes in Okazaki fragment strandedness observed in human initiation zones set up the need for broadly distributed potential start sites that may exceed the number of ORC binding sites. Overall, in agreement with pioneering studies of the DHFR locus ^55, 60^, these results indicate that in metazoan cells, the entire genome is a potential substrate for stochastic initiation but the efficiency of individual sites is epigenetically and developmentally regulated, in coordination with transcriptional activity.

## Replisome assembly at eukaryotic DNA replication origins and disassembly at termination sites

The control of replisome assembly at eukaryotic replication origins relies on a strict temporal separation of replicative helicase loading and activation ( Figure 1b) ^61, 62^. From late mitosis to the late G1-phase of the cell cycle, the ORC together with Cdc6, another AAA+ ATPase, and Cdt1 load the ring-shaped, Mcm2-7 replicative helicase motor in the form of a catalytically inactive head-to-head dimer around double-stranded DNA (dsDNA). This process, called origin licensing or pre-replicative complex (preRC) assembly, has been reconstituted with purified budding yeast proteins ^63, 64^. Origins are then activated during S-phase, which is triggered by a rise in Clb5,6- and Dbf4-dependent protein kinase (CDK and DDK) activities ^65^. In this complex process, the Mcm2-7 double hexamer (MCM DH) associates with helicase cofactors Cdc45 and GINS and a number of other initiation factors ^65^, resulting in the formation of two active Cdc45/Mcm2-7/GINS (CMG) holo-helicases ^66^ that encircle ssDNA ^67^ and seed replisome assembly. The head-to-head configuration of the MCM DH thus provides a molecular mechanism for the establishment of bidirectional DNA synthesis at eukaryotic origins. Once cells enter the S-phase, several well-studied mechanisms prevent *de novo* MCM loading onto origins ^68^. More elusive are the mechanisms that eliminate unfired MCMs from chromatin as DNA synthesis proceeds ^69^. Recent work shows that when converging forks meet and terminate replication, converging CMGs pass one another and leading and lagging strands are rapidly ligated ^70^, then CMGs are disassembled by ATPase p97 following ubiquitylation of MCM7 ^71, 72^. Passage through mitosis and G1-phase is then required for a new round of origin licensing prior to DNA replication.

A remarkable recent achievement is the reconstitution of helicase loading followed by helicase activation and DNA synthesis with purified budding yeast proteins (16 factors made of 42 polypeptides) ^73^. Even though DNA synthesis in this system does not yet fully recapitulate normal leading and lagging strand replication, this work defines the minimum set of factors required for origin-dependent replication initiation and sets the stage for complete reconstitution of chromosome replication. Other important recent achievements are a crystal structure of the *Drosophila* ORC ^74^ and a cryo-EM, near atomic structure of the MCM DH purified from yeast G1 chromatin ^75^. The ORC structure suggests that ORC can switch between autoinhibited and active conformations, exposing a gap in the ORC ring where DNA can bind and be trapped by joining of Cdc6 prior to MCM loading. In the MCM DH structure, the two single hexamers are twisted and tilted to form a kinked central channel. The kink, located at the DH interface, is proposed to deform DNA and act as a nucleation center for DNA unwinding. DDK-dependent opening of the rings at the MCM2-MCM5 interface may create an expanded central chamber for strand separation through which ssDNA may loop out and become accessible to the copying process.

## Mcm2-7 DHs are loaded in excess to origins

In contrast to the bacterial mechanism of replication initiation, neither the binding of ORC to DNA nor the loading of the MCM DH during G1 results in any detectable ssDNA formation ( Figure 1) ^76^. Furthermore, once MCM DHs are loaded, ORC, Cdc6, and Cdt1 are no longer required for replication initiation ^73, 77, 78^. The CMG helicase, whose assembly is restricted to S-phase, is solely responsible for DNA unwinding at origins and at moving forks. Therefore, activated MCM DHs, not ORC, ultimately determine where replication can initiate.

**Figure 3.**
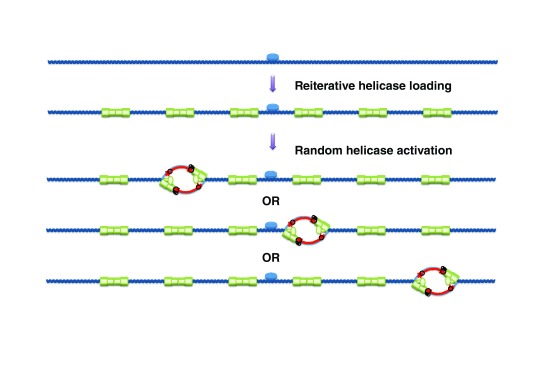
Model for broad replication initiation zones in metazoan cells. During origin licensing, multiple Mcm2-7 double hexamers (MCM DHs) are loaded onto a large region surrounding the origin recognition complex (ORC). Only a small fraction of MCM DHs is then activated in any one S-phase. Thus, initiation can potentially occur at any of a large number of sites in a broad zone around the ORC.

Importantly, studies in budding yeast ^79^ and metazoans ^80– 82^ revealed that MCM proteins are bound to chromatin in G1-phase at levels that far exceed (by a factor of 10 to 50) the number of active replication origins and ORC. This raises questions about the loading mechanism and the location and function of these abundant MCMs. Early experiments with model DNA substrates in *Xenopus* egg extracts ^83– 85^ suggested that many copies of MCM could be loaded, and initiate DNA replication, over a large region around ORC, suggesting a mechanism for the formation of broad initiation zones from a single ORC ( Figure 3). A single MCM DH encircles ∼60 bp of dsDNA ^63^. Presumably, MCMs loaded next to the ORC would need to spread through the surrounding chromatin to liberate space for reiterated MCM loading. *In vitro* reconstitution of yeast origin licensing has shown that MCM DHs can passively slide along dsDNA ^63^, which may facilitate their spreading around the ORC. The punctate nature of budding yeast replication initiation sites suggests that if MCM spreading also occurs in yeast, ORC-proximal MCMs are favored for initiation, which is in contrast to the situation in *Xenopus*.

Spreading of MCM DHs along chromosome arms is expected to require the displacement of nucleosomes, as the channel in the MCM DH is not wide enough to accommodate nucleosomal particles. Genome-wide measurements of nucleosome turnover in *Drosophila* cells suggest that turnover is high around ORC binding sites and proportional to ORC binding ^86^. High nucleosome turnover may facilitate ORC binding, and perhaps MCM loading and spreading around the ORC. Among the many factors reported to promote preRC assembly, the histone H4 acetylase HBO1 ^87^, the ATP-dependent chromatin remodeler SNF2H ^88^, and the novel histone-binding protein GRWD1 ^89^, which are all recruited to chromatin by interaction with Cdt1, have been proposed to promote MCM loading in human cells. One possible model is that H4 acetylation by HBO1 promotes the recruitment of SNF2H and/or GRWD1, which cooperatively increase chromatin fluidity to facilitate MCM loading.

It may be less necessary to invoke MCM spreading if ORC can bind at multiple locations throughout broad initiation zones. Metazoan ORC binds weakly and cycles on and off DNA quickly ^90^, whereas MCM DHs are highly stable once loaded ^69^, which may explain the large excess of MCM in relation to ORC on G1 chromatin. However, human genome-wide analysis suggests that ORC binding sites, unlike initiation sites, are not uniformly distributed through initiation zones but concentrate at their borders ^59^, consistent with ORC preference for the promoters of active genes ^91^. Furthermore, the broad initiation zones reconstituted in *Xenopus* experiments do reflect MCM spreading from ORC, since DNA hypermethylation was used to prevent ORC binding through the template except in a small, low-CpG-density DNA region ^84^.

In *Xenopus*, the redundancy of potential origins afforded by excess MCMs has been proposed to ensure a reliable S-phase completion time by allowing initiation to increase late in S-phase inside long inter-origin gaps ^85, 92, 93^. A comparable time-dependent increase in initiation rate has been observed in widely divergent eukaryotes and proposed as a universal feature of S-phase kinetics ^94^. When MCMs are depleted by up to 90%, although S-phase progression is not obviously altered, progressive accumulation of DNA damage is observed ^95^. This genotoxic effect is strongly potentiated by treatment with drugs that slow fork progression. Cells from several metazoan organisms can activate extra origins to maintain a normal rate of S-phase progression in response to fork slowing, but this response is abolished when MCMs are depleted by >90% ^95– 97^. However, clear evidence for a similar role of excess MCMs as back-up origins has not been reported in budding yeast.

## Single and reiterative MCM DH loading

In nuclear extracts derived from G1-arrested yeast cells, the ORC can load at least four MCM complexes on a 1 kb fragment containing a single copy of the early firing origin *ARS1* ^98^. Importantly, an *orc 4* mutation that blocks ATP hydrolysis (but not ATP binding) by the ORC allows a single round of MCM loading in this system ^98^. ATP hydrolysis by the ORC is therefore required for reiterated MCM loading. According to a recent study of origin licensing using real-time, single molecule imaging of fluorescently labeled ORCs, MCMs, and origin DNA, the ORC is released from the origin after the assembly of a single MCM DH ^99^. If so, the ORC would need to cycle on and off origin DNA to load multiple MCM DHs. Conceivably, ATP hydrolysis may facilitate ORC release from loaded MCM DHs and ADP/ATP exchange would reactivate the ORC for another round of loading. Reiterative loading was detected only at a high concentration of purified proteins in reconstitution experiments ^63, 99^ and was far less efficient than with extracts ^98^. Extracts may contain factors that promote ADP/ATP exchange by the ORC or that actively push loaded MCM DHs along dsDNA to liberate space for repeated MCM loading at the same site.

## Reiterative MCM DH loading and replication timing regulation

**Figure 4.**
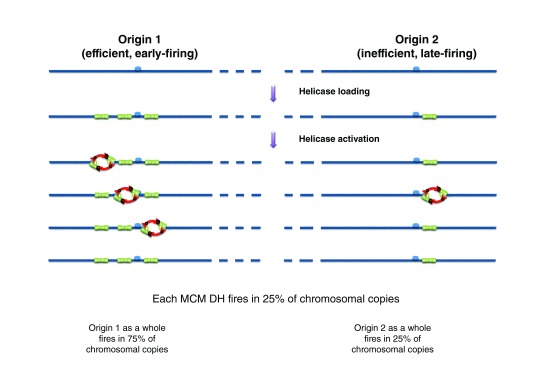
The multiple-MCM model for regulating origin firing efficiency and time. If a variable number of Mcm2-7 double hexamers (MCM DHs) are loaded at origins and each MCM DH has a constant probability of firing per unit time, origins with more MCM DHs have as a whole a higher probability of firing and an earlier mean firing time.

One possible function of excess MCM loading is in regulating origin firing time. A computational analysis of genome-wide replication kinetics in budding yeast shows that earlier-firing origins have a tighter firing time distribution and a higher potential efficiency than later-firing origins ^31^. Furthermore, analysis of several ChIP-seq experiments suggests that MCM peaks are on average stronger at early origins than at late origins ^100^, although no such correlation between ORC or MCM levels and origin efficiency was observed in another study ^33^ that used different ChIP-seq datasets ^101^. The observed correlation suggests a model in which origins fire stochastically but are loaded with a variable number of MCMs so that origins that have more MCMs fire on average earlier and at a more precise time than origins with fewer MCMs ( Figure 4) ^31^. Recent quantitative ORC and MCM Western blots on purified plasmid origins detected, on average, three MCM DHs at early origin *ARS1*, two at early origin *ARS305* but fewer than one at late origin *ARS306* ^100^. Finally, an *ARS1* mutation that reduced MCM loading 6-fold without affecting ORC binding strongly delayed *ARS1* firing time ^100^. These results support the multiple-MCM model for replication timing regulation.

ORC occupancy, chromatin context, and a number of trans-acting factors might all determine MCM multiplicity at origins, and some of these factors may also regulate origin firing time by affecting the activation of MCMs after they have been loaded ^102^. In *Drosophila*, ORC-rich origins show a higher rate of nucleosome exchange than ORC-poor origins ^86^. In yeast, early origins have a wider NFR, and a higher occupancy and better positioning of adjacent nucleosomes, than late origins ^103^. Mutations in chromatin remodelers and histone-modifying enzymes might be expected to affect MCM spreading and consequent origin efficiency. However, correlative evidence suggests that the Rpd3 histone deacetylase, the KU telomere binding protein, the Fkh1 transcription factor, or the Ctf19 kinetochore protein, which all affect replication timing by modifying chromatin structure in yeast, do so independently of MCM number ^100^. The ATP-dependent chromatin remodeling complexes Isw2 and Ino80, which promote yeast DNA replication specifically in the late-replicating regions, apparently do so by facilitating replication fork progression but not late origin firing ^104^. Therefore, chromatin remodelers that specifically increase MCM spreading and multiplicity in budding yeast remain to be identified. Rbr1, the yeast homolog of GRWD1, a histone-binding protein that facilitates MCM loading in human cells, is a possible candidate ^89^.

## Fine mapping of MCM DHs and initiation sites at ARSs

The multiple-MCM model predicts multiple potential initiation sites at early origins ( Figure 3), in apparent conflict with the identification of a single start site for each leading strand within the NFR of *ARS1* ^44^. However, sequences outside the NFR were not examined in the mapping of *ARS1* initiation sites. A recent genome-wide study, in apparent conflict with both the multiple-MCM model and the fine mapping of leading strand start sites at *ARS1*, suggests that a single MCM DH is loaded per origin but not at the NFR ^105^. In this work, micrococcal nuclease (MNase) footprinting of wild-type and *orc1* mutant cells reveals ORC-dependent footprints at one-half of all (800) putative origins previously identified in a plasmid assay ^106^. ORC footprints extend downstream from the ACS into the NFR and are surrounded by well-positioned nucleosomes. When MNase footprints are compared with ChIP-seq data, ORC is found to coincide with the ACS, as expected. However, MCMs do not map to the NFR but to either the upstream or the downstream nucleosome, with which they likely form a complex protecting a total of ∼210 bp of DNA. Genome-wide mapping of replication initiation sites at nucleotide resolution would be required to further evaluate whether they coincide with ARS NFRs or flanking nucleosomes. Although a single MCM DH per origin is detected in these experiments ^105^, additional MCM DHs may escape detection if they are not complexed with nucleosomes and translocate off DNA during MNase digestion or if they are too heterogeneously scattered to form ChIP-seq peaks. These results may thus be reconciled with the large body of evidence for an excess of chromatin-bound MCMs to ORCs.

## Dispersive MCM DH loading and non-canonical budding yeast origins

In ChIP-seq experiments, most MCM peaks coincide with ORC peaks and with ARSs ^35, 100, 101, 107^. However, it seems difficult to account for the 10–20-fold excess of MCMs to ORCs in G1-phase yeast chromatin ^79^ by the close packing of 5–10 MCM DHs in the immediate vicinity of each ARS. Only two or three MCM DHs were observed *in vivo* at plasmid-borne *ARS305* and *ARS1* ^100^. Therefore, the ChIP-seq peak signal at origins may represent only a fraction of loaded MCMs while the rest may be too heterogeneously spread to form detectable peaks. It is also possible that, at steady state, ORC is bound to only a fraction of ARS because it cycles on and off rapidly. Having 3 MCM DHs and 0.3 ORCs per ARS would give an MCM/ORC ratio of 20. However, ORC occupancy at ARS1 has been reported to be high *in vivo* ^42^.

In principle, yeast MCMs may spread from the ORC over large distances, as demonstrated in human cells ^108^, in *Xenopus* egg extracts ^83^, and recently in *Drosophila* cells ^82^. The spreading mechanism is unknown. An ORC bound to an ARS may load MCMs at distal sites by chromosomal looping. However, a DNA loop is not obviously compatible with the coaxial alignment of ORC and MCM rings observed in origin licensing intermediates ^109^. Alternatively, MCMs loaded next to an ORC may spread through chromatin by nucleosome displacement and remodeling. Finally, the ORC may occasionally bind DNA and load MCMs elsewhere than at ARSs. *In vitro* experiments show that the yeast ORC can direct functional MCM DH loading on plasmid DNA devoid of ARSs ^78, 110^. Since ARSs are strictly required for plasmid maintenance *in vivo*, but are dispensable *in vitro* for replication of DNA not occluded by nucleosomes, one may speculate that *in vivo* the ORC sometimes binds and loads MCM DHs opportunistically at NFRs not associated with ARSs.

It was reported years ago that a yeast chromosome III derivative entirely devoid of ARS elements still replicates and segregates correctly 97% of the time and that the ORC is required for its maintenance ^111^. The location of the putative non-canonical initiation events responsible for maintenance was not determined. Recently, an origin-deficient derivative of yeast chromosome VI was also found to replicate robustly, and initiation was observed at non-canonical loci located around deleted origins ^112^. The ability to direct replication from non-canonical sites in an ORC-dependent manner is consistent with the loading of MCM DHs elsewhere than at ARSs. Further work is required to evaluate the prevalence of non-canonical initiation events in normal S-phase as well as in conditions of replicative stress and their effect on yeast chromosomal replication robustness.

## MCM spreading by transcription

**Figure 5.**
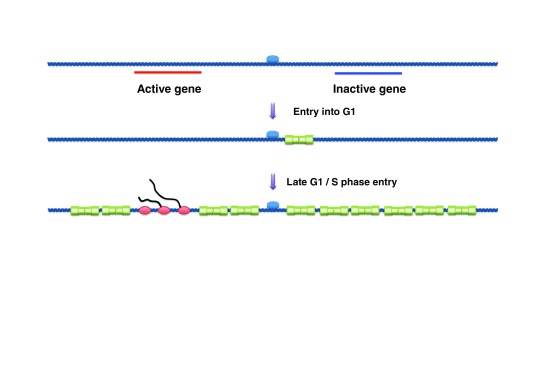
Dynamic loading and redistribution of Mcm2-7 double hexamers (MCM DHs) during the G1-phase in metazoans. In early G1, MCM DHs are loaded in small numbers and colocalize with the origin recognition complex (ORC). MCM DH loading increases during the course of G1-phase. At the G1/S transition, a biphasic pattern of MCM DH binding is observed, with broad chromosomal regions containing MCM DHs punctuated by exclusion of MCM DHs from transcribed regions.

Recently, MCM loading and distribution have been quantified at different points in the cell cycle of *Drosophila* Kc cells ^82^. This important work provides the first genome-wide view of MCM distribution in a higher eukaryote and reveals a dramatic reorganization of MCMs during late G1 ( Figure 5). As expected, cells arrested at the G1/S transition with hydroxyurea (HU) had a robust accumulation of MCMs on chromatin. However, ∼10-fold fewer MCMs were loaded in cells arrested in late G1 by overexpression of Dacapo, a cyclin E/Cdk2 inhibitor, or by RNAi against cyclin E or Cdk2. When Dacapo-arrested cells were released back into the cell cycle, a ∼10-fold increase in MCM chromatin association was observed coincident with entry into S-phase. Both the early and late G1-phases of MCM loading required Cdc6 and Cdt1, a signature of the canonical origin licensing pathway.

ChIP-chip was used to localize ORCs in asynchronous cells and MCMs in G1 or G1/S-arrested cells. ORCs and MCMs in cyclin E RNAi-treated cells were highly concordant with each other and localized to early origins. In HU-arrested cells, in contrast, a binary pattern of broad, MCM-containing chromosomal regions alternating with MCM-free regions was observed through the genome. Therefore, the full complement of MCMs was loaded and redistributed throughout the genome in late G1/early S ( Figure 5). Active genes had no or very little MCM signal, whereas inactive genes and intergenic DNA exhibited an elevated signal. When two cell lines were compared, genes active in only one cell line were depleted of MCMs only in that cell line. Although it is plausible that HU-stalled forks contributed to MCM redistribution, the transcription-dependent, biphasic pattern of MCM binding was observed at both early and non-early origins. Therefore, MCMs loaded in late G1 are displaced from transcribed genes by active transcription and cannot be re-established or translocate in these regions after the G1/S transition ( Figure 5). These results ^82^ fit nicely with the widespread detection of broad replication initiation zones bounded by active transcription units in human cells ^59^.

**Figure 6.**
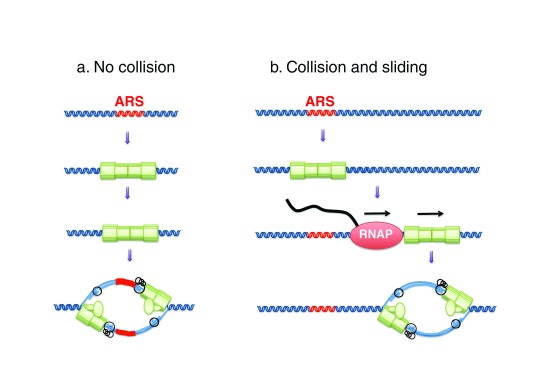
Transcription through a licensed origin shifts the position of initiation sites. Mcm2-7 double hexamers (MCM DHs) loaded at a licensed origin respond to collisions with transcription complexes by sliding along the template and remain functional for replication initiation at their new location. ARS, autonomously replicating sequence; RNAP, RNA polymerase.

A recent study in budding yeast has analyzed how MCM DHs respond to collisions with transcription complexes ( Figure 6) ^113^. MCM DHs were reconstituted on an ARS plasmid carrying a T7 RNA polymerase (RNAP) promoter. In the presence of T7 transcription, MCM DHs remained stably bound to circular but not linear DNA molecules. Therefore, T7 RNAP did not disassemble the MCM DHs but pushed them off the ends of the DNA. Importantly, T7 RNAP transcription did not interfere with the ability of circular templates to replicate in S-phase extracts. However, MCM DHs and initiation sites were shifted by up to several kbp. Given that many yeast origins are located downstream of protein-coding genes, the effect of RNAP collisions with origins was examined *in vivo* in yeast cells harboring a thermosensitive mutation in the transcription termination factor Rat1. After two hours of asynchronous growth at non-permissive temperature, ORC ChIP-seq peaks were not altered, whereas MCM peaks were broadened and shifted by up to a few kbp in the direction of transcription. Okazaki fragment sequencing revealed that frequent shifts in origin position correlated with the shift in MCM distribution. Loss of origin efficiency was also observed, which could be due to several factors including 1) dispersive origin redistribution, 2) displacement of MCM DHs at sites not permissive for initiation, or 3) prevention of origin licensing by invading transcription. Collisions between RNAP and MCM DHs may contribute to the displacement of MCMs from transcription units in late G1 ^82^ and the establishment of replication initiation zones bounded by active transcription units in higher eukaryotes ^59^.

These recent results highlight the passive spreading of MCM DHs along chromosome arms by the transcription machinery as a mechanism to specify replication initiation sites on eukaryotic chromosomes. A similar mechanism has been previously proposed for the relocation of the ring-shaped cohesin complex along yeast chromosomes ^114^. Differently from MCM DHs, however, cohesin rings appear not to be displaced but traversed by the replisome, which results in cohesion between nascent sister chromatids. What distinguishes the replisome from the transcription apparatus so that it does not push cohesin, but may slide through it, is unclear. Might cohesin assist MCM spreading? Cohesin loading is independent of preRC proteins in budding yeast ^114^, colocalizes with ORC independently of other preRC proteins in *Drosophila* ^115^, and is dependent on chromatin-bound MCMs in *Xenopus* egg extracts ^116, 117^. Given that cohesin appears to have functions beyond sister chromatid cohesion by entrapping DNA segments of the same chromosome ^118^, it is interesting to consider, in addition to collision and pushing by RNAP, that MCM DHs may be loaded away from ORC via cohesin-mediated chromatin looping.

## Conclusions and perspectives

There is increasing evidence that the spreading of multiple MCM DHs around the ORC, as first reported in metazoan cells, also occurs in budding yeast and that MCM multiplicity regulates origin firing time and safeguards the genome against incomplete replication. Multiple mechanisms that involve various DNA translocation and nucleosome eviction machineries probably contribute to this spreading. MCM DHs indeed respond to collisions with transcription complexes by sliding along the template yet remain functional for replication initiation, which partly explains how transcription programs shape the replication landscape of metazoan cells.

As mentioned above, the mechanisms that eliminate unfired MCM DHs from chromatin as DNA synthesis proceeds are unclear. Do replication forks collide with unfired MCM DHs or are such collisions avoided by anticipated removal of MCM DHs ahead of forks? Removing MCM DHs in advance of such collisions seems counterproductive, as it would deplete unreplicated DNA segments from backup origins and increase their vulnerability to fork stalling. If collisions occur, are collided MCM DHs disassembled, or are they pushed ahead of the elongating replisome to serve as "portable" rescue origins? No doubt the coming years will bring answers to some of these fascinating questions.

## References

[1] DePamphilis ML Bell SD: Genome Duplication. Garland Science,2011 Reference Source

[2] Hyrien O: Peaks cloaked in the mist: the landscape of mammalian replication origins. *J Cell Biol.* 2015;208(2):147–60. 10.1083/jcb.201407004 25601401 PMC4298691

[3] Blumenthal AB Kriegstein HJ Hogness DS: The units of DNA replication in *Drosophila melanogaster* chromosomes. *Cold Spring Harb Symp Quant Biol.* 1974;38:205–23. 10.1101/SQB.1974.038.01.024 4208784

[4] Callan HG: Replication of DNA in the chromosomes of eukaryotes. *Proc R Soc Lond B Biol Sci.* 1972;181(1062):19–41. 4402332 10.1098/rspb.1972.0039

[5] Hyrien O Maric C Méchali M: Transition in specification of embryonic metazoan DNA replication origins. *Science.* 1995;270(5238):994–7. 10.1126/science.270.5238.994 7481806

[6] Sasaki T Sawado T Yamaguchi M: Specification of regions of DNA replication initiation during embryogenesis in the 65-kilobase *DNApolalpha-dE2F* locus of *Drosophila melanogaster*. *Mol Cell Biol.* 1999;19(1):547–55. 10.1128/MCB.19.1.547 9858578 PMC83912

[7] Norio P Kosiyatrakul S Yang Q: Progressive activation of DNA replication initiation in large domains of the immunoglobulin heavy chain locus during B cell development. *Mol Cell.* 2005;20(4):575–87. 10.1016/j.molcel.2005.10.029 16307921

[8] Göndör A Ohlsson R: Replication timing and epigenetic reprogramming of gene expression: a two-way relationship? *Nat Rev Genet.* 2009;10(4):269–76. 10.1038/nrg2555 19274048

[9] Ahuja AK Jodkowska K Teloni F: A short G1 phase imposes constitutive replication stress and fork remodelling in mouse embryonic stem cells. *Nat Commun.* 2016;7:10660. 10.1038/ncomms10660 26876348 PMC4756311

[10] Ruiz S Lopez-Contreras AJ Gabut M: Limiting replication stress during somatic cell reprogramming reduces genomic instability in induced pluripotent stem cells. *Nat Commun.* 2015;6:8036. 10.1038/ncomms9036 26292731 PMC4560784

[11] Muñoz S Méndez J: DNA replication stress: from molecular mechanisms to human disease. *Chromosoma.* 2016;1–15. 10.1007/s00412-016-0573-x 26797216

[12] Berti M Vindigni A: Replication stress: getting back on track. *Nat Struct Mol Biol.* 2016;23(2):103–9. 10.1038/nsmb.3163 26840898 PMC5125612

[13] Mirkin EV Mirkin SM: To switch or not to switch: at the origin of repeat expansion disease. *Mol Cell.* 2014;53(1):1–3. 10.1016/j.molcel.2013.12.021 24411078 PMC3940269

[14] Jackson AP Laskey RA Coleman N: Replication proteins and human disease. *Cold Spring Harb Perspect Biol.* 2014;6(1): pii: a013060. 10.1101/cshperspect.a013060 23881941 PMC3941220

[15] Dobbelstein M Sørensen CS: Exploiting replicative stress to treat cancer. *Nat Rev Drug Discov.* 2015;14(6):405–23. 10.1038/nrd4553 25953507

[16] Robinson A Causer RJ Dixon NE: Architecture and conservation of the bacterial DNA replication machinery, an underexploited drug target. *Curr Drug Targets.* 2012;13(3):352–72. 10.2174/138945012799424598 22206257 PMC3290774

[17] Naeem A Badshah SL Muska M: The Current Case of Quinolones: Synthetic Approaches and Antibacterial Activity. *Molecules.* 2016;21(4):268. 10.3390/molecules21040268 27043501 PMC6274096

[18] Berdis AJ: DNA polymerases as therapeutic targets. *Biochemistry.* 2008;47(32):8253–60. 10.1021/bi801179f 18642851 PMC2692436

[19] Leonard AC Grimwade JE: The orisome: structure and function. *Front Microbiol.* 2015;6:545. 10.3389/fmicb.2015.00545 26082765 PMC4451416

[20] Kelman LM Kelman Z: Archaeal DNA replication. *Annu Rev Genet.* 2014;48:71–97. 10.1146/annurev-genet-120213-092148 25421597

[21] Huberman JA Riggs AD: On the mechanism of DNA replication in mammalian chromosomes. *J Mol Biol.* 1968;32(2):327–41. 10.1016/0022-2836(68)90013-2 5689363

[22] Hyrien O: Up and Down the Slope: Replication Timing and Fork Directionality Gradients in Eukaryotic Genomes.In *The Initiation of DNA Replication in Eukaryotes* (ed D Kaplan) Ch. 4, (Springer).2016;65–85. 10.1007/978-3-319-24696-3_4

[23] Stinchcomb DT Struhl K Davis RW: Isolation and characterisation of a yeast chromosomal replicator. *Nature.* 1979;282(5734):39–43. 10.1038/282039a0 388229

[24] Struhl K Stinchcomb DT Scherer S: High-frequency transformation of yeast: autonomous replication of hybrid DNA molecules. *Proc Natl Acad Sci U S A.* 1979;76(3):1035–9. 10.1073/pnas.76.3.1035 375221 PMC383183

[25] Chan CS Tye BK: Autonomously replicating sequences in Saccharomyces cerevisiae. *Proc Natl Acad Sci U S A.* 1980;77(11):6329–33. 10.1073/pnas.77.11.6329 7005897 PMC350277

[26] Brewer BJ Fangman WL: The localization of replication origins on ARS plasmids in S. cerevisiae. *Cell.* 1987;51(3):463–71. 10.1016/0092-8674(87)90642-8 2822257

[27] Huberman JA Spotila LD Nawotka KA: The *in vivo* replication origin of the yeast 2 microns plasmid. *Cell.* 1987;51(3):473–81. 10.1016/0092-8674(87)90643-X 3311385

[28] Fangman WL Brewer BJ: Activation of replication origins within yeast chromosomes. *Annu Rev Cell Biol.* 1991;7:375–402. 10.1146/annurev.cb.07.110191.002111 1809350

[29] Raghuraman MK Winzeler EA Collingwood D: Replication dynamics of the yeast genome. *Science.* 2001;294(5540):115–21. 10.1126/science.294.5540.115 11588253

[30] Raghuraman MK Brewer BJ: Molecular analysis of the replication program in unicellular model organisms. *Chromosome Res.* 2010;18(1):19–34. 10.1007/s10577-009-9099-x 20012185 PMC3976475

[31] Yang SC Rhind N Bechhoefer J: Modeling genome-wide replication kinetics reveals a mechanism for regulation of replication timing. *Mol Syst Biol.* 2010;6:404. 10.1038/msb.2010.61 20739926 PMC2950085

[32] Hawkins M Retkute R Müller CA: High-resolution replication profiles define the stochastic nature of genome replication initiation and termination. *Cell Rep.* 2013;5(4):1132–41. 10.1016/j.celrep.2013.10.014 24210825 PMC3898788

[33] McGuffee SR Smith DJ Whitehouse I: Quantitative, genome-wide analysis of eukaryotic replication initiation and termination. *Mol Cell.* 2013;50(1):123–35. 10.1016/j.molcel.2013.03.004 23562327 PMC3628276

[34] Muller CA Hawkins M Retkute R: The dynamics of genome replication using deep sequencing. *Nucleic Acids Res.* 2014;42(1):e3. 10.1093/nar/gkt878 24089142 PMC3874191

[35] Eaton ML Galani K Kang S: Conserved nucleosome positioning defines replication origins. *Genes Dev.* 2010;24(8):748–53. 10.1101/gad.1913210 20351051 PMC2854390

[36] Newlon CS Theis JF: The structure and function of yeast *ARS* elements. *Curr Opin Genet Dev.* 1993;3(5):752–8. 10.1016/S0959-437X(05)80094-2 8274858

[37] Bell SP Stillman B: ATP-dependent recognition of eukaryotic origins of DNA replication by a multiprotein complex. *Nature.* 1992;357(6374):128–34. 10.1038/357128a0 1579162

[38] Bell SP Kobayashi R Stillman B: Yeast origin recognition complex functions in transcription silencing and DNA replication. *Science.* 1993;262(5141):1844–9. 10.1126/science.8266072 8266072

[39] Foss M McNally FJ Laurenson P: Origin recognition complex (ORC) in transcriptional silencing and DNA replication in *S. cerevisiae*. *Science.* 1993;262(5141):1838–44. 10.1126/science.8266071 8266071

[40] Micklem G Rowley A Harwood J: Yeast origin recognition complex is involved in DNA replication and transcriptional silencing. *Nature.* 1993;366(6450):87–9. 10.1038/366087a0 8232543

[41] Diffley JF Cocker JH: Protein-DNA interactions at a yeast replication origin. *Nature.* 1992;357(6374):169–72. 10.1038/357169a0 1579168

[42] Diffley JF Cocker JH Dowell SJ: Two steps in the assembly of complexes at yeast replication origins *in vivo*. *Cell.* 1994;78(2):303–16. 10.1016/0092-8674(94)90299-2 8044842

[43] Carpenter PB Mueller PR Dunphy WG: Role for a *Xenopus* Orc2-related protein in controlling DNA replication. *Nature.* 1996;379(6563):357–60. 10.1038/379357a0 8552193

[44] Bielinsky AK Gerbi SA: Chromosomal *ARS1* has a single leading strand start site. *Mol Cell.* 1999;3(4):477–86. 10.1016/S1097-2765(00)80475-X 10230400

[45] Harland RM Laskey RA: Regulated replication of DNA microinjected into eggs of Xenopus laevis. *Cell.* 1980;21(3):761–71. 10.1016/0092-8674(80)90439-0 6254667

[46] Hyrien O Méchali M: Plasmid replication in *Xenopus* eggs and egg extracts: a 2D gel electrophoretic analysis. *Nucleic Acids Res.* 1992;20(7):1463–9. 10.1093/nar/20.7.1463 1349740 PMC312223

[47] Mahbubani HM Paull T Elder JK: DNA replication initiates at multiple sites on plasmid DNA in Xenopus egg extracts. *Nucleic Acids Res.* 1992;20(7):1457–62. 10.1093/nar/20.7.1457 1579437 PMC312222

[48] Krysan PJ Haase SB Calos MP: Isolation of human sequences that replicate autonomously in human cells. *Mol Cell Biol.* 1989;9(3):1026–33. 10.1128/MCB.9.3.1026 2542763 PMC362692

[49] Krysan PJ Calos MP: Replication initiates at multiple locations on an autonomously replicating plasmid in human cells. *Mol Cell Biol.* 1991;11(3):1464–72. 10.1128/MCB.11.3.1464 1996103 PMC369425

[50] Remus D Beall EL Botchan MR: DNA topology, not DNA sequence, is a critical determinant for *Drosophila* ORC-DNA binding. *EMBO J.* 2004;23(4):897–907. 10.1038/sj.emboj.7600077 14765124 PMC380993

[51] Vashee S Cvetic C Lu W: Sequence-independent DNA binding and replication initiation by the human origin recognition complex. *Genes Dev.* 2003;17(15):1894–908. 10.1101/gad.1084203 12897055 PMC196240

[52] Schaarschmidt D Baltin J Stehle IM: An episomal mammalian replicon: sequence-independent binding of the origin recognition complex. *EMBO J.* 2004;23(1):191–201. 10.1038/sj.emboj.7600029 14685267 PMC1271667

[53] Shinomiya T Ina S: Analysis of chromosomal replicons in early embryos of *Drosophila melanogaster* by two-dimensional gel electrophoresis. *Nucleic Acids Res.* 1991;19(14):3935–41. 10.1093/nar/19.14.3935 1907366 PMC328486

[54] Hyrien O Méchali M: Chromosomal replication initiates and terminates at random sequences but at regular intervals in the ribosomal DNA of *Xenopus* early embryos. *EMBO J.* 1993;12(12):4511–20. 8223461 10.1002/j.1460-2075.1993.tb06140.x PMC413880

[55] Hamlin JL Mesner LD Dijkwel PA: A winding road to origin discovery. *Chromosome Res.* 2010;18(1):45–61. 10.1007/s10577-009-9089-z 19859818 PMC2904547

[56] Vaughn JP Dijkwel PA Hamlin JL: Replication initiates in a broad zone in the amplified CHO dihydrofolate reductase domain. *Cell.* 1990;61(6):1075–87. 10.1016/0092-8674(90)90071-L 2350784

[57] Dijkwel PA Hamlin JL: The Chinese hamster dihydrofolate reductase origin consists of multiple potential nascent-strand start sites. *Mol Cell Biol.* 1995;15(6):3023–31. 10.1128/MCB.15.6.3023 7760799 PMC230533

[58] Dijkwel PA Mesner LD Levenson VV: Dispersive initiation of replication in the Chinese hamster rhodopsin locus. *Exp Cell Res.* 2000;256(1):150–7. 10.1006/excr.2000.4809 10739662

[59] Petryk N Kahli M d'Aubenton-Carafa Y: Replication landscape of the human genome. *Nat Commun.* 2016;7: 10208. 10.1038/ncomms10208 26751768 PMC4729899

[60] Mesner LD Li X Dijkwel PA: The dihydrofolate reductase origin of replication does not contain any nonredundant genetic elements required for origin activity. *Mol Cell Biol.* 2003;23(3):804–14. 10.1128/MCB.23.3.804-814.2003 12529386 PMC140713

[61] Deegan TD Diffley JF: MCM: one ring to rule them all. *Curr Opin Struct Biol.* 2016;37:145–51. 10.1016/j.sbi.2016.01.014 26866665

[62] Remus D Diffley JF: Eukaryotic DNA replication control: lock and load, then fire. *Curr Opin Cell Biol.* 2009;21(6):771–7. 10.1016/j.ceb.2009.08.002 19767190

[63] Remus D Beuron F Tolun G: Concerted loading of Mcm2-7 double hexamers around DNA during DNA replication origin licensing. *Cell.* 2009;139(4):719–30. 10.1016/j.cell.2009.10.015 19896182 PMC2804858

[64] Evrin C Clarke P Zech J: A double-hexameric MCM2-7 complex is loaded onto origin DNA during licensing of eukaryotic DNA replication. *Proc Natl Acad Sci U S A.* 2009;106(48):20240–5. 10.1073/pnas.0911500106 19910535 PMC2787165

[65] Tognetti S Riera A Speck C: Switch on the engine: how the eukaryotic replicative helicase MCM2-7 becomes activated. *Chromosoma.* 2015;124(1):13–26. 10.1007/s00412-014-0489-2 25308420

[66] Ilves I Petojevic T Pesavento JJ: Activation of the MCM2-7 helicase by association with Cdc45 and GINS proteins. *Mol Cell.* 2010;37(2):247–58. 10.1016/j.molcel.2009.12.030 20122406 PMC6396293

[67] Fu YV Yardimci H Long DT: Selective bypass of a lagging strand roadblock by the eukaryotic replicative DNA helicase. *Cell.* 2011;146(6):931–41. 10.1016/j.cell.2011.07.045 21925316 PMC3209622

[68] Siddiqui K On KF Diffley JF: Regulating DNA replication in eukarya. *Cold Spring Harb Perspect Biol.* 2013;5(9): pii: a012930. 10.1101/cshperspect.a012930 23838438 PMC3753713

[69] Kuipers MA Stasevich TJ Sasaki T: Highly stable loading of Mcm proteins onto chromatin in living cells requires replication to unload. *J Cell Biol.* 2011;192(1):29–41. 10.1083/jcb.201007111 21220507 PMC3019549

[70] Dewar JM Budzowska M Walter JC: The mechanism of DNA replication termination in vertebrates. *Nature.* 2015;525(7569):345–50. 10.1038/nature14887 26322582 PMC4575634

[71] Maric M Maculins T De Piccoli G: Cdc48 and a ubiquitin ligase drive disassembly of the CMG helicase at the end of DNA replication. *Science.* 2014;346(6208):1253596. 10.1126/science.1253596 25342810 PMC4300516

[72] Moreno SP Bailey R Campion N: Polyubiquitylation drives replisome disassembly at the termination of DNA replication. *Science.* 2014;346(6208):477–81. 10.1126/science.1253585 25342805

[73] Yeeles JT Deegan TD Janska A: Regulated eukaryotic DNA replication origin firing with purified proteins. *Nature.* 2015;519(7544):431–5. 10.1038/nature14285 25739503 PMC4874468

[74] Bleichert F Botchan MR Berger JM: Crystal structure of the eukaryotic origin recognition complex. *Nature.* 2015;519(7543):321–6. 10.1038/nature14239 25762138 PMC4368505

[75] Li N Zhai Y Zhang Y: Structure of the eukaryotic MCM complex at 3.8 Å. *Nature.* 2015;524(7564):186–91. 10.1038/nature14685 26222030

[76] Geraghty DS Ding M Heintz NH: Premature structural changes at replication origins in a yeast minichromosome maintenance (MCM) mutant. *J Biol Chem.* 2000;275(24):18011–21. 10.1074/jbc.M909787199 10751424

[77] Hua XH Newport J: Identification of a preinitiation step in DNA replication that is independent of origin recognition complex and cdc6, but dependent on cdk2. *J Cell Biol.* 1998;140(2):271–81. 10.1083/jcb.140.2.271 9442103 PMC2132576

[78] Gros J Devbhandari S Remus D: Origin plasticity during budding yeast DNA replication *in vitro*. *EMBO J.* 2014;33(6):621–36. 10.1002/embj.201387278 24566988 PMC3989655

[79] Donovan S Harwood J Drury LS: Cdc6p-dependent loading of Mcm proteins onto pre-replicative chromatin in budding yeast. *Proc Natl Acad Sci U S A.* 1997;94(11):5611–6. 10.1073/pnas.94.11.5611 9159120 PMC20826

[80] Mahbubani HM Chong JP Chevalier S: Cell cycle regulation of the replication licensing system: involvement of a Cdk-dependent inhibitor. *J Cell Biol.* 1997;136(1):125–35. 10.1083/jcb.136.1.125 9008708 PMC2132454

[81] Burkhart R Schulte D Hu D: Interactions of human nuclear proteins P1Mcm3 and P1Cdc46. *Eur J Biochem.* 1995;228(2):431–8. 10.1111/j.1432-1033.1995.0431n.x 7705359

[82] Powell SK MacAlpine HK Prinz JA: Dynamic loading and redistribution of the Mcm2–7 helicase complex through the cell cycle. *EMBO J.* 2015;34(4):531–43. 10.15252/embj.201488307 25555795 PMC4331006

[83] Edwards MC Tutter AV Cvetic C: MCM2–7 complexes bind chromatin in a distributed pattern surrounding the origin recognition complex in *Xenopus* egg extracts. *J Biol Chem.* 2002;277(36):33049–57. 10.1074/jbc.M204438200 12087101

[84] Harvey KJ Newport J: CpG methylation of DNA restricts prereplication complex assembly in *Xenopus* egg extracts. *Mol Cell Biol.* 2003;23(19):6769–79. 10.1128/MCB.23.19.6769-6779.2003 12972597 PMC193934

[85] Lucas I Chevrier-Miller M Sogo JM: Mechanisms ensuring rapid and complete DNA replication despite random initiation in *Xenopus* early embryos. *J Mol Biol.* 2000;296(3):769–86. 10.1006/jmbi.2000.3500 10677280

[86] Deal RB Henikoff JG Henikoff S: Genome-wide kinetics of nucleosome turnover determined by metabolic labeling of histones. *Science.* 2010;328(5982):1161–4. 10.1126/science.1186777 20508129 PMC2879085

[87] Miotto B Struhl K: HBO1 histone acetylase activity is essential for DNA replication licensing and inhibited by Geminin. *Mol Cell.* 2010;37(1):57–66. 10.1016/j.molcel.2009.12.012 20129055 PMC2818871

[88] Sugimoto N Yugawa T Iizuka M: Chromatin remodeler sucrose nonfermenting 2 homolog (SNF2H) is recruited onto DNA replication origins through interaction with Cdc10 protein-dependent transcript 1 (Cdt1) and promotes pre-replication complex formation. *J Biol Chem.* 2011;286(45):39200–10. 10.1074/jbc.M111.256123 21937426 PMC3234745

[89] Sugimoto N Maehara K Yoshida K: Cdt1–binding protein GRWD1 is a novel histone-binding protein that facilitates MCM loading through its influence on chromatin architecture. *Nucleic Acids Res.* 2015;43(12):5898–911. 10.1093/nar/gkv509 25990725 PMC4499137

[90] Sonneville R Querenet M Craig A: The dynamics of replication licensing in live *Caenorhabditis* elegans embryos. *J Cell Biol.* 2012;196(2):233–46. 10.1083/jcb.201110080 22249291 PMC3265957

[91] Dellino GI Cittaro D Piccioni R: Genome-wide mapping of human DNA-replication origins: levels of transcription at ORC1 sites regulate origin selection and replication timing. *Genome Res.* 2013;23(1):1–11. 10.1101/gr.142331.112 23187890 PMC3530669

[92] Herrick J Stanislawski P Hyrien O: Replication fork density increases during DNA synthesis in *X. laevis* egg extracts. *J Mol Biol.* 2000;300(5):1133–42. 10.1006/jmbi.2000.3930 10903859

[93] Hyrien O Marheineke K Goldar A: Paradoxes of eukaryotic DNA replication: MCM proteins and the random completion problem. *Bioessays.* 2003;25(2):116–25. 10.1002/bies.10208 12539237

[94] Goldar A Marsolier-Kergoat MC Hyrien O: Universal temporal profile of replication origin activation in eukaryotes. *PLoS One.* 2009;4(6):e5899. 10.1371/journal.pone.0005899 19521533 PMC2690853

[95] Ibarra A Schwob E Méndez J: Excess MCM proteins protect human cells from replicative stress by licensing backup origins of replication. *Proc Natl Acad Sci U S A.* 2008;105(26):8956–61. 10.1073/pnas.0803978105 18579778 PMC2449346

[96] Woodward AM Göhler T Luciani MG: Excess Mcm2-7 license dormant origins of replication that can be used under conditions of replicative stress. *J Cell Biol.* 2006;173(5):673–83. 10.1083/jcb.200602108 16754955 PMC2063885

[97] Ge XQ Jackson DA Blow JJ: Dormant origins licensed by excess Mcm2-7 are required for human cells to survive replicative stress. *Genes Dev.* 2007;21(24):3331–41. 10.1101/gad.457807 18079179 PMC2113033

[98] Bowers JL Randell JC Chen S: ATP hydrolysis by ORC catalyzes reiterative Mcm2-7 assembly at a defined origin of replication. *Mol Cell.* 2004;16(6):967–78. 10.1016/j.molcel.2004.11.038 15610739

[99] Ticau S Friedman LJ Ivica NA: Single-molecule studies of origin licensing reveal mechanisms ensuring bidirectional helicase loading. *Cell.* 2015;161(3):513–25. 10.1016/j.cell.2015.03.012 25892223 PMC4445235

[100] Das SP Borrman T Liu VW: Replication timing is regulated by the number of MCMs loaded at origins. *Genome Res.* 2015;25(12):1886–92. 10.1101/gr.195305.115 26359232 PMC4665009

[101] Xu W Aparicio JG Aparicio OM: Genome-wide mapping of ORC and Mcm2p binding sites on tiling arrays and identification of essential ARS consensus sequences in *S. cerevisiae*. *BMC Genomics.* 2006;7:276. 10.1186/1471-2164-7-276 17067396 PMC1657020

[102] Das SP Rhind N: How and why multiple MCMs are loaded at origins of DNA replication. *Bioessays.* 2016;38(7):613–7. 10.1002/bies.201600012 27174869 PMC5052224

[103] Soriano I Morafraile EC Vázquez E: Different nucleosomal architectures at early and late replicating origins in *Saccharomyces cerevisiae.* *BMC Genomics.* 2014;15:791. 10.1186/1471-2164-15-791 25218085 PMC4176565

[104] Vincent JA Kwong TJ Tsukiyama T: ATP-dependent chromatin remodeling shapes the DNA replication landscape. *Nat Struct Mol Biol.* 2008;15(5):477–84. 10.1038/nsmb.1419 18408730 PMC2678716

[105] Belsky JA MacAlpine HK Lubelsky Y: Genome-wide chromatin footprinting reveals changes in replication origin architecture induced by pre-RC assembly. *Genes Dev.* 2015;29(2):212–24. 10.1101/gad.247924.114 25593310 PMC4298139

[106] Nieduszynski CA Hiraga S Ak P: OriDB: a DNA replication origin database. *Nucleic Acids Res.* 2007;35(Database issue):D40–6. 10.1093/nar/gkl758 17065467 PMC1781122

[107] Wyrick JJ Aparicio JG Chen T: Genome-wide distribution of ORC and MCM proteins in *S. cerevisiae*: high-resolution mapping of replication origins. *Science.* 2001;294(5550):2357–60. 10.1126/science.1066101 11743203

[108] Ritzi M Baack M Musahl C: Human minichromosome maintenance proteins and human origin recognition complex 2 protein on chromatin. *J Biol Chem.* 1998;273(38):24543–9. 10.1074/jbc.273.38.24543 9733749

[109] Sun J Fernandez-Cid A Riera A: Structural and mechanistic insights into Mcm2-7 double-hexamer assembly and function. *Genes Dev.* 2014;28(20):2291–303. 10.1101/gad.242313.114 25319829 PMC4201289

[110] On KF Beuron F Frith D: Prereplicative complexes assembled *in vitro* support origin-dependent and independent DNA replication. *EMBO J.* 2014;33(6):605–20. 10.1002/embj.201387369 24566989 PMC3989654

[111] Dershowitz A Snyder M Sbia M: Linear derivatives of *Saccharomyces cerevisiae* chromosome III can be maintained in the absence of autonomously replicating sequence elements. *Mol Cell Biol.* 2007;27(13):4652–63. 10.1128/MCB.01246-06 17452442 PMC1951491

[112] Bogenschutz NL Rodriguez J Tsukiyama T: Initiation of DNA replication from non-canonical sites on an origin-depleted chromosome. *PLoS One.* 2014;9(12):e114545. 10.1371/journal.pone.0114545 25486280 PMC4259332

[113] Gros J Kumar C Lynch G: Post-licensing Specification of Eukaryotic Replication Origins by Facilitated Mcm2-7 Sliding along DNA. *Mol Cell.* 2015;60(5):797–807. 10.1016/j.molcel.2015.10.022 26656162 PMC4680849

[114] Lengronne A Katou Y Mori S: Cohesin relocation from sites of chromosomal loading to places of convergent transcription. *Nature.* 2004;430(6999):573–8. 10.1038/nature02742 15229615 PMC2610358

[115] MacAlpine HK Gordan R Powell SK: *Drosophila* ORC localizes to open chromatin and marks sites of cohesin complex loading. *Genome Res.* 2010;20(2):201–11. 10.1101/gr.097873.109 19996087 PMC2813476

[116] Gillespie PJ Hirano T: Scc2 couples replication licensing to sister chromatid cohesion in *Xenopus* egg extracts. *Curr Biol.* 2004;14(17):1598–603. 10.1016/j.cub.2004.07.053 15341749

[117] Takahashi TS Yiu P Chou MF: Recruitment of Xenopus Scc2 and cohesin to chromatin requires the pre-replication complex. *Nat Cell Biol.* 2004;6(10):991–6. 10.1038/ncb1177 15448702

[118] Losada A: Cohesin in cancer: chromosome segregation and beyond. *Nat Rev Cancer.* 2014;14(6):389–93. 10.1038/nrc3743 24854081

[119] Smith DJ Whitehouse I: Intrinsic coupling of lagging-strand synthesis to chromatin assembly. *Nature.* 2012;483(7390):434–8. 10.1038/nature10895 22419157 PMC3490407

